# Evaluation of Biological and Sociodemographic Factors Affecting Dysmenorrhea

**DOI:** 10.7759/cureus.7977

**Published:** 2020-05-05

**Authors:** Ayse Cetin

**Affiliations:** 1 Emergency Medicine, Liv Hospital Istanbul, Istanbul, TUR

**Keywords:** dysmenorrhea, menarche, menstruation, pelvic pain, pain severity

## Abstract

Objective

Dysmenorrhea is the most important cause of chronic pelvic pain in women. Sometimes, dysmenorrhea can be severe enough, leading women to present to emergency departments. The aim of this study was to investigate factors affecting dysmenorrhea in female patients who presented to the emergency department of our hospital.

Methods

Female patients who presented to our emergency department with dysmenorrhea between January 2012 and January 2014 were included in the study. Patients’ demographic and clinical data were filled in the Dysmenorrhea Data Form, which was designed by the researcher by screening the relevant literature. Patients’ age, educational status, smoking status, age and regularity of menarche, sexual activity, and age of dysmenorrhea onset were recorded and analyzed.

Results

The mean age of the patients was 21.80 ± 3.75 years. There was a significant correlation between the type of dysmenorrhea and sexual activity (p=0.001). There was a statistically significant difference between age at menarche and age of dysmenorrhea onset (p<0.001). Absenteeism was less common in patients with an age of dysmenorrhea onset of <12 years compared with the other age groups (p<0.05).

Conclusions

There was a significant correlation between age at menarche and age of dysmenorrhea onset. Data obtained in this study could be used in developing educational programs on dysmenorrhea for adolescents at the age of menarche.

## Introduction

Dysmenorrhea, one of the most common causes of pelvic pain and menstrual disorders, is defined as uterine-induced painful cramps. Dysmenorrhea is a severe pain localized primarily in the lower abdominal quadrant, resembling labor pain. This pain can be defined as a cramp-like severe or blunt pain, suggesting compression in the pelvic region [1]. The majority of women report that they experience moderate-to-severe symptoms such as nausea, vomiting, headache, diarrhea, fatigue, irritability, dizziness, and syncope with dysmenorrhea [2]. Dysmenorrhea is the leading cause of gynecologic morbidity regardless of age [3]. According to the World Health Organization, dysmenorrhea is the most important cause of chronic pelvic pain [4]. When studies on the prevalence of dysmenorrhea were examined, the incidence was reported between 45.3% and 90% [5]. Although dysmenorrhea is not a life-threatening condition, it may affect the quality of life in women and may cause disability if it is severe enough. Also, dysmenorrhea leads to mental problems in some women, causing them to be isolated and avoid participation in different social activities [6].

Dysmenorrhea can be divided into two categories: primary (spasmodic) and secondary (congestive). Primary dysmenorrhea is defined as menstrual pain without macroscopic pelvic pathology involved. Normally, it occurs within the first several weeks after menarche. In primary dysmenorrhea, pain is usually localized in the suprapubic region in the form of cramps or spasm. Pain begins with menstruation and ends within 48-72 hours. Secondary dysmenorrhea is defined as menstrual pain resulting from anatomical or macroscopic pelvic pathologies. Pain may begin before menstruation and last a few days after menstruation [7].

Several studies have defined the risk factors of severe dysmenorrhea episodes. These may include smoking, an earlier age at menarche, positive family history, prolonged menstrual periods, obesity, and alcohol consumption [8,9]. Depression and stress have also been shown to increase the risk of dysmenorrhea [10].

Various pharmacological and nonpharmacological methods are used in the management of dysmenorrhea, including non-steroidal anti-inflammatory drugs (NSAIDs), herbal remedies, diet therapies, yoga, meditation, and acupuncture [11].

Sometimes, dysmenorrhea can be severe enough to result in women presenting to emergency departments. It is important to determine the factors affecting dysmenorrhea, which is an important public health problem. In this study, we aimed to investigate the effects of various biological and sociodemographic factors on dysmenorrhea in female patients who presented to the emergency department of our hospital due to dysmenorrhea.

## Materials and methods

Female patients who presented to our emergency department with concerns of dysmenorrhea and completed a data form between January 2012 and January 2014 were included in the study. Patients who presented to the emergency department with concerns of abdominal pain due to other reasons, those who had entered menopause and had pain, those who presented with dysmenorrhea-like complaints but were diagnosed with different abdominal pain, and those who did not agree to complete a data form were excluded from the study. Patients’ demographic and clinical data were filled in the Dysmenorrhea Data Form, which was designed by the researcher by screening the relevant literature. Patients’ age, educational status, height, weight, marital status, smoking status, age and regularity of menarche, sexual activity, age of dysmenorrhea onset, time and duration of dysmenorrhea onset, additional symptoms (nausea, vomiting, diarrhea, headache), and absenteeism were recorded in the Dysmenorrhea Data Form. In addition, patients' analgesic use, analgesics used (NSAID, paracetamol, etc.), hormonal therapy, history of systemic diseases, and previous operations were also recorded in the forms. Based on the responses given by the patients, those with underlying disease were categorized as secondary dysmenorrhea, and patients without underlying disease were categorized as primary dysmenorrhea. Pain severity of the patients was measured using a descriptive category scale. Accordingly, patients were asked to score their pain severity between 1 and 10 points. Based on the responses, the cases were grouped as mild if <4, moderate if 5-8, and severe if 9-10 points.

Ethics statement

Prior to beginning, the study protocol was approved by the Local Ethics Committee of our hospital. All patients were informed about the objectives of the studies, and they provided their consent. The study was conducted in line with the ethical principles of the Declaration of Helsinki.

Statistical analysis

Data obtained from the study were statistically analyzed using SPSS Statistics for Windows, Version 17.0 (SPSS Inc., Chicago, IL, USA). Descriptive statistics were expressed as number and percentage. Numerical variables were expressed as mean ± standard deviation and as minimum and maximum values. Normality of the variables was tested with the Kolmogorov-Smirnov test. Mann-Whitney U and Kruskal-Wallis tests were used to analyze continuous variables. Categorical variables were analyzed using chi-square and Fisher’s exact tests, and p < 0.05 values were considered statistically significant.

## Results

A total of 215 female patients who presented to the emergency department of our hospital with the concern of dysmenorrhea between January 2012 and January 2014 were included in the study. The mean age of the patients was 21.80 ± 3.75 years (minimum-maximum: 16-45 years). Of all patients, 84.7% (n=182) were college graduates. When patients were evaluated according to body mass index (BMI) values, 20.9% (n=45) were weak, 69.8% (n=150) were normal, 7% (n=15) were overweight, and 2.3% (n=5) were obese. Of the patients, 94% (n=202) were single and 30.2% (n=65) were smokers. Menstruation was regular in 66.5% (n=143) and irregular in 33.5% (n=72) of patients. Of the patients, 12.6% (n=27) were receiving hormonal therapy. While 17.3% (n=37) of the patients had a history of previous surgery, 6.5% (n=14) had systemic diseases.

When patients were evaluated according to the time of pain onset, pain began before menstruation in 50.2% (n=108) and during menstruation in 49.8% (n=107). Of all patients, 9.8% (n=21) reported that they were sexually active. Among all dysmenorrhea cases, pain onset was more commonly before menstruation in the presence of sexual activity (81%) and during menstruation in the absence of sexual activity (53.1%), and the difference was statistically significant (p<0.05). In addition, there was a statistically significant correlation between the time of pain onset and educational status (p=0.029).

Primary dysmenorrhea was found in 80% (n=172) and secondary dysmenorrhea in 20% (n=43) of the patients. There was a significant correlation between the type of dysmenorrhea and sexual activity (p=0.001).

**Figure 1:**
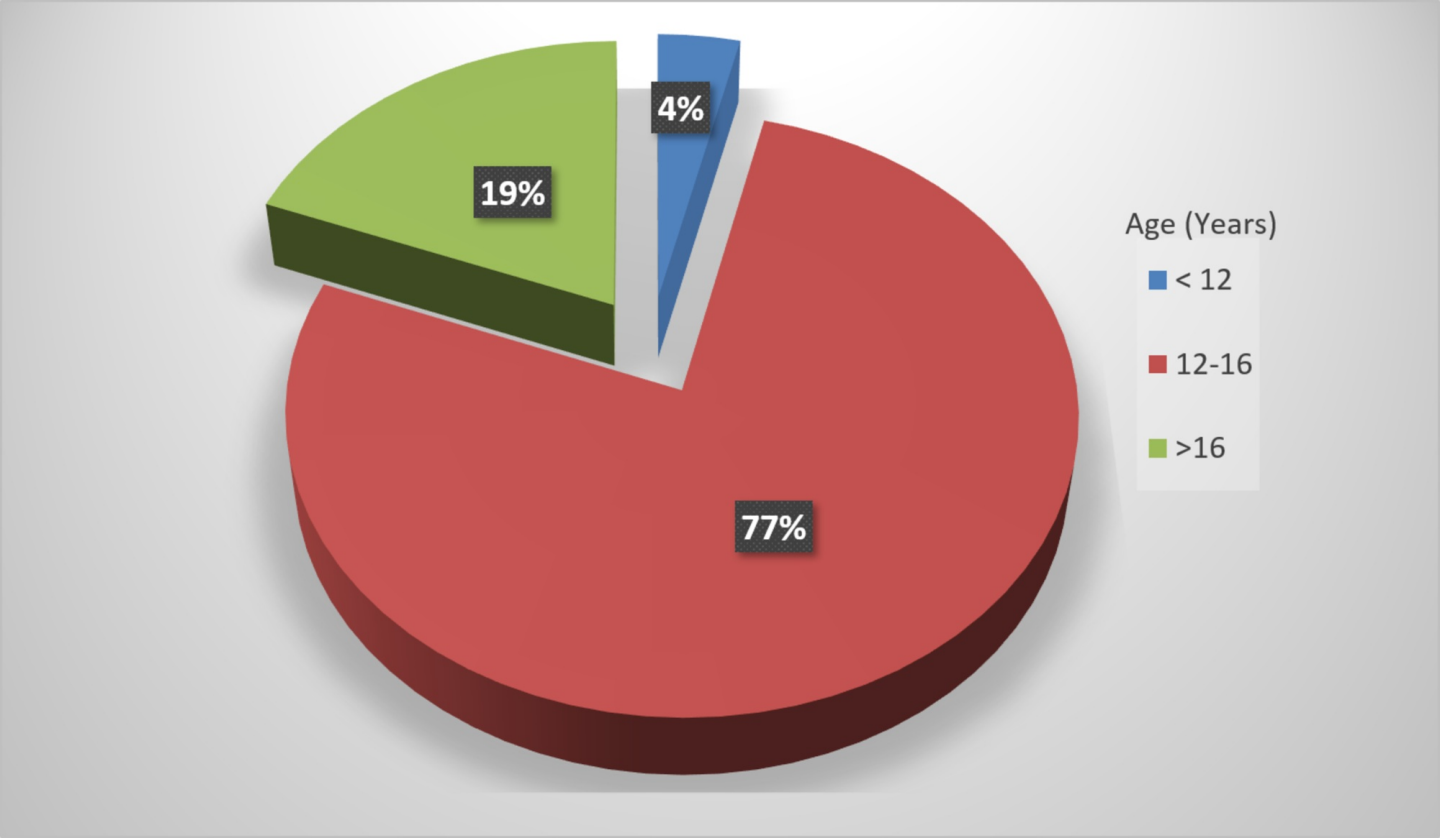
Distribution of age ranges at menarche

The mean age at menarche was 13.37 ± 1.38 years. The mean age of dysmenorrhea onset was 14.68 ± 3.47 years. Of all patients, 3.7% (n=8) reported that dysmenorrhea began before 12 years of age, 77.2% (n=166) between 12 and 16 years of age, and 19.1% (n=41) after 16 years of age (Figure 1). There was a statistically significant difference between age at menarche and age of dysmenorrhea onset (p<0.001). Dysmenorrhea was associated with secondary causes in 16.3% (n=35) of the patients. In these patients, the etiologies of dysmenorrhea are given in Table 1. A statistically significant correlation was found between secondary causes and BMI (p=0.028).

**Table 1:** Etiology of secondary dysmenorrhea cases

	Number of Cases	% of Cases
Primary dysmenorrhea	172	80.0
Secondary dysmenorrhea	43	20.0
Etiology		
Ovarian cyst	28	13.0
Endometriosis	4	1.9
Myoma	2	0.9
Endometrial polyps	1	0.5

While dysmenorrhea caused absenteeism in 74.9% (n=161), it did not cause absenteeism in 25.1% (n=54). It was found that absenteeism was less common in patients with an age of pain onset of <12 years compared with the other age groups (<12: 37.5%; 12-16: 75.9%; >16: 78%), and the difference was statistically significant (p<0.05).

**Figure 2:**
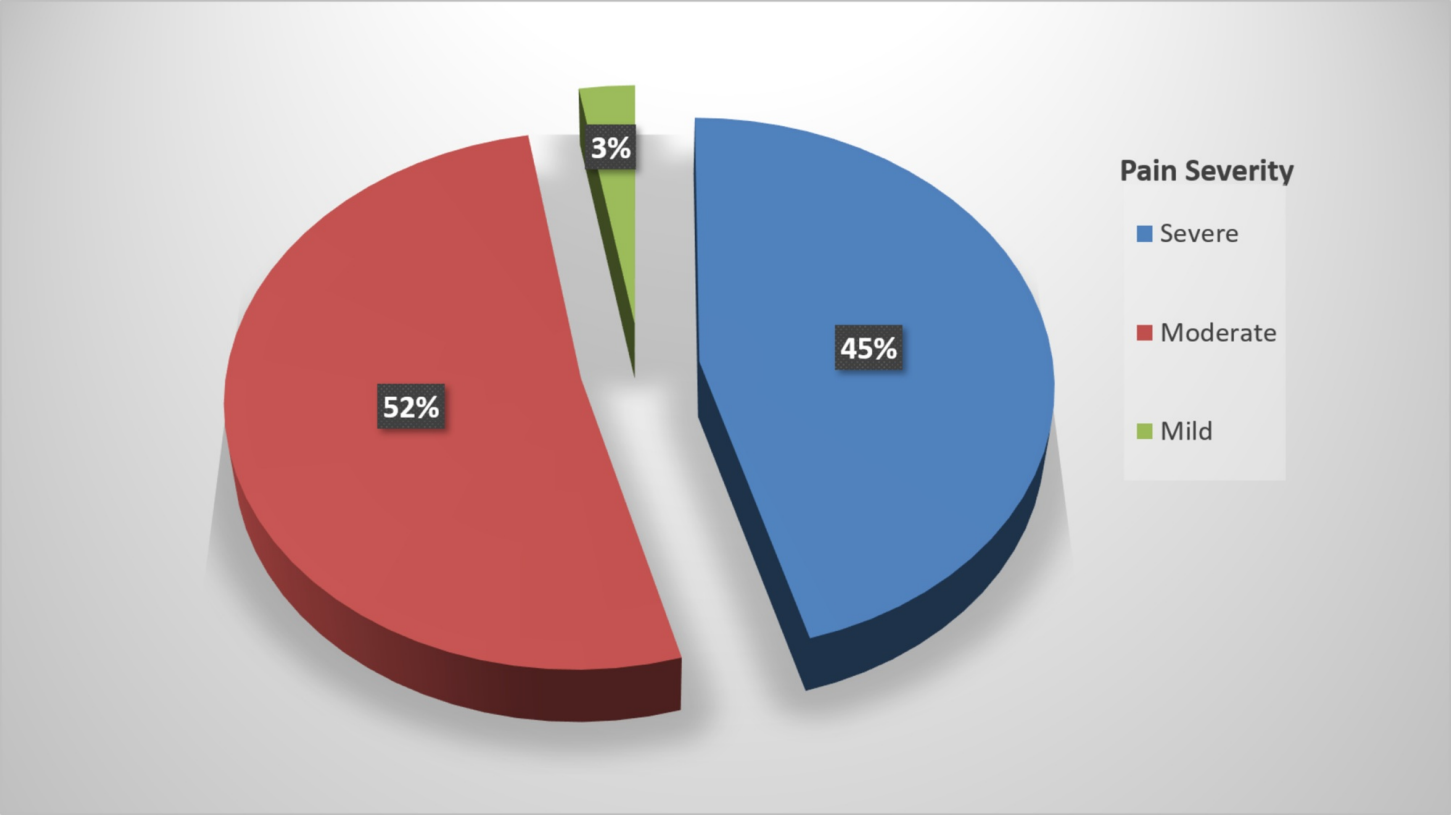
Pain severity scores according to descriptive category scale reported by the patients

Patients were asked to score their pain from 1 to 10 points. Accordingly, pain severity was grouped as severe in 45.6% (n=98), moderate in 51.6% (n=111), and mild in 2.8% (n=6). There was a significant correlation between pain severity and sexual activity (p=0.001; Figure 2).

When the patients were evaluated in terms of the accompanying symptoms, additional symptoms (nausea, vomiting, headache, diarrhea, etc.) were found in 79.1% (n=170). A statistically significant correlation was found between the presence of additional symptoms accompanying dysmenorrhea and smoking (p=0.049).

Of all patients, 81.4% (n=175) reported that they used analgesics. Among these patients, 74.2% (n=130) reported that they were receiving NSAIDs, 14.9% (n=26) were receiving paracetamol, and 10.9% (n=19) were receiving smooth muscle relaxants. There was a statistically significant correlation between the use of analgesics and educational status (p=0.012).

Of the 202 patients who responded to the question “Did you benefit from pain killers?”, 57.9% (n=117) stated that they benefited, whereas 42.1% (n=85) reported no benefit. There was a statistically significant correlation between the status of benefiting from drugs and educational status (p<0.001).

In our study, no statistically significant correlation was found between BMI, marital status, smoking, menstruation regularity, and absenteeism, and the other examined parameters.

## Discussion

In this investigating the effects of several biological and sociodemographic factors on dysmenorrhea in patients who presented to the emergency department with concerns of dysmenorrhea, in addition to other factors, we studied the effects of smoking status, sexual activity, educational status, menstrual regularity, age at menarche, and accompanying symptoms. Smoking has been shown to be among the primary risk factors of dysmenorrhea in the literature [8,9]. In a study by Dorn et al. from the United States, the effect of smoking on menstrual symptoms was examined, and it was revealed that smoking increased the incidence of primary dysmenorrhea. In the aforementioned study, 60.7% of participants were smokers [12]. In a meta-analysis by Jenabi et al. in 2018, 14 studies investigating the relationship between smoking and dysmenorrhea were reviewed, and it was found that all of these studies reported a significant correlation between smoking status and dysmenorrhea [13]. However, data on this issue are conflicting in the literature. In a study by Fernández-Martínez et al. of female college students in Spain, it was reported that smoking was not a factor increasing the risk of dysmenorrhea [14]. Similarly, in our study, no statistically significant correlation was found between smoking and primary dysmenorrhea. However, the relatively lower rate of smokers (30.2%) among our patients compared with the other studies might contribute to these findings.

In addition, studies have also examined the effect of smoking on the severity of dysmenorrhea symptoms. In a study by Burnett et al. from Canada, female smokers were found to have more severe dysmenorrhea symptoms [15]. In our study, smoking status and dysmenorrhea pain severity were compared, and no significant difference was determined (p>0.05).

In this study, the mean age at menarche was determined to be 13.37 ± 1.38 years, and the mean age of dysmenorrhea onset was 14.68 ± 3.47 years. There was a statistically significant correlation between age at menarche and age of dysmenorrhea onset (p<0.001). In a study by Muluneh et al. in North-West Ethiopia in 2018, early age at menarche was associated with an increase in the prevalence of dysmenorrhea [16]. Akbarzadeh et al. reported a significant association between age at menarche and dysmenorrhea onset [17]. On the other hand, Kural et al. could not find such a correlation [18]. The different results may be attributed to the differences between nutritional habits, public health, geographic location, and cultural factors among the studies. According to the literature, dysmenorrhea usually begins within one to two years after menarche [19]. This indicates the importance of providing adolescent girls at the age of menarche with education about dysmenorrhea. In our study, dysmenorrhea began in the 12- to 16-year-old age group in 77.2% of the patients. This finding is consistent with the results previously reported in the literature [20].

In a study by Chen and Chen from the United States, adolescents were observed to largely have moderate-to-severe menstrual cramps [21]. In our study, 45.6% of the patients described their pain as severe, 51.6% as moderate, and 2.8% as mild. The reason for more common moderate-to-severe pain might be due to patients with mild pain not commonly presenting to the emergency department. In a study by Gun et al., dysmenorrhea began with menstruation in 39.9%, one to two hours before menstruation in 37.2%, and a few days prior to menstruation in 22.9% of the participants [22]. In a study by Erenel and Şentürk of students, dysmenorrhea began with menstruation in 38.4%, two to three days before menstruation in 29.3%, and one to two hours before menstruation in 26.3% [23]. According to our findings, dysmenorrhea began before menstruation in 50.2% and during menstruation in 49.8% of the patients. In addition, time of dysmenorrhea onset was statistically significantly correlated with educational status and sexual activity. Dysmenorrhea onset was before menstruation in the presence of sexual activity (81%), and the difference was statistically significant (p<0.05). It can be thought that as the educational level increases, pain is more correctly described. Hormonal therapies used for contraceptive purposes may affect time of dysmenorrhea onset.

In a study by Latthe et al. investigating the factors causing chronic pelvic pain in women, BMI < 20 kg/m2 was reported as a risk factor for primary dysmenorrhea [4]. Harlow and Campbell found a significant correlation between obesity and dysmenorrhea and reported that pain was more than twofold in obese patients compared with others [3]. In our study, we could not determine a significant correlation between BMI values and dysmenorrhea. However, the number of obese patients was very low (2.3%) in our study.

Similarly, in a study by Unsal et al. investigating the prevalence of dysmenorrhea and its effects on quality of life, no significant correlation was found between obesity and dysmenorrhea among college students [24]. Again, Fujiwara and Nakata from Japan reported no significant association between BMI and dysmenorrhea among Japanese college students [25]. Our result is similar to those from the studies by Unsal et al. and Fujiwara et al. [24,25]. This may be due to the patient population in both the mentioned studies and our study mainly consisting of young students who may care about body image and weight and are more likely to have a BMI value within the normal range.

Study limitations

This study has some limitations. Because our hospital was located near a college, the majority of cases presenting due to dysmenorrhea consisted of students. College students more commonly have primary dysmenorrhea. This resulted in a small number of cases with secondary dysmenorrhea. Furthermore, the number of patients with mild pain was low. These patients might have determined a therapy method by themselves without presenting to the emergency department.

## Conclusions

In this study, there was a significant correlation between age at menarche and age of dysmenorrhea onset. Severe pain was found in patients with an age of dysmenorrhea onset of <12 years. Significant correlations were found between pain severity and sexual activity and between the type of dysmenorrhea and sexual activity. No significant correlation was found between BMI values, smoking, and dysmenorrhea. The obtained data could be used in developing educational programs on dysmenorrhea for adolescents at the age of menarche.
